# Development of DNA Aptamers to Native EpCAM for Isolation of Lung Circulating Tumor Cells from Human Blood

**DOI:** 10.3390/cancers11030351

**Published:** 2019-03-12

**Authors:** Galina S. Zamay, Olga S. Kolovskaya, Tatiana I. Ivanchenko, Tatiana N. Zamay, Dmitry V. Veprintsev, Valentina L. Grigorieva, Irina I. Garanzha, Alexey V. Krat, Yury E. Glazyrin, Ana Gargaun, Ivan N. Lapin, Valery A. Svetlichnyi, Maxim V. Berezovski, Anna S. Kichkailo

**Affiliations:** 1 Federal Research Center “Krasnoyarsk Science Center of the Siberian Branch of the Russian Academy of Science”, Krasnoyarsk 660036, Russia; galina.zamay@gmail.com (G.S.Z.); olga.kolovskaya@gmail.com (O.S.K.); cobweb9999@gmail.com (T.I.I.); tzamay@yandex.ru (T.N.Z.); d_veprintsev@mail.ru (D.V.V.); yury.glazyrin@gmail.com (Y.E.G.); 2 Laboratory for Biomolecular and Medical Technologies, Krasnoyarsk State Medical University named after prof. V.F. Voino-Yasenecki, Krasnoyarsk 660022, Russia; valllya93@mail.ru (V.L.G.); garanzha.i.v@mail.ru (I.I.G.); alexkrat@mail.ru (A.V.K.); 3 Krasnoyarsk Regional Clinical Cancer Center named after A.I. Kryzhanovsky, Krasnoyarsk 660133, Russia; 4 Department of Chemistry and Biomolecular Sciences, University of Ottawa, Ottawa, ON K1N 6N5, Canada; ana.gargaun@gmail.com; 5 Laboratory of Advanced Materials and Technology, Siberian Physical-Technical Institute of Tomsk State University, Tomsk 634050, Russia; 201kiop@mail.ru (I.N.L.); v_svetlichnyi@bk.ru (V.A.S.)

**Keywords:** aptamers, SELEX (Systematic evolution of ligands by exponential enrichment), EpCAM, non-small-cell lung cancer, circulating tumor cells, blood

## Abstract

We selected DNA aptamers to the epithelial cell adhesion molecule (EpCAM) expressed on primary lung cancer cells isolated from the tumors of patients with non-small cell lung cancer using competitive displacement of aptamers from EpCAM by a corresponding antibody. The resulting aptamers clones showed good nanomolar affinity to EpCAM-positive lung cancer cells. Confocal microscopy imaging and spectral profiling of lung cancer tissues confirmed the same protein target for the aptamers and anti-EpCAM antibodies. Furthermore, the resulted aptamers were successfully applied for isolation and detection of circulating tumor cells in clinical samples of peripheral blood of lung cancer patients.

## 1. Introduction

Monoclonal antibodies (mAbs) are one of the current success stories of modern biotechnology. Hundreds of different diagnostic kits based on mAb technology are available in the market [1,2,3]. The key advantage of mAbs is their high specificity for the relevant disease targets, which limits harm to healthy cells, resulting in fewer adverse effects compared to traditional pharmaceuticals based on small organic molecules. However, early excitement has been tempered by problems associated with the use of mAbs. These large (150 kDa) multimeric proteins contain numerous disulphide bonds and posttranslational modifications such as glycosylation, and require a sophisticated eukaryotic machinery to be produced in an active form [3]. The fragment crystallizable region (Fc) of mAbs can interact with Fc receptors expressed at the surface of many cell types, which increases their cross-reactivity and retention in the circulation. Moreover, mAbs developed in animals must be humanized prior to use in clinical applications. Consequently, the production of therapeutic mAbs necessitates use of very large cultures of mammalian cells followed by extensive purification under good manufacturing practice (GMP) conditions. Overall, high production costs, regulatory affairs, safety and short shelf life have thus limited broad use of these antibodies.

These limitations have sparked considerable interest in the development of molecular tools that offer the functionality of mAbs, but that can be easily engineered, synthesized, and tailored to match desired characteristics. Aptamers have enormous potential as such a tool [4,5]. These small (5–30 kDa), single-stranded DNA and RNA molecules carry the blueprint for their own synthesis in their primary sequence, so they can be synthesized by chemical or enzymatic procedures. They fold into well-defined three-dimensional structures, show high affinity and specificity for their targets, and can be used to inhibit biological functions. Aptamers are often viewed as “chemical antibodies” with applications ranging from target detection to drug discovery and delivery. In many respects, aptamers are superior to antibodies. They can be chemically synthesized in high purity at low cost (thousands of times cheaper than mAbs), and are considered as a synthetic chemical product, but not a biological product. Biological products are produced from or extracted from a biological (living) system and require, in addition to physico-chemical testing, biological testing for full characterization. Lastly, aptamers are low-immunogenic and low-toxic and can be applied as drugs themselves [5,6,7]. Indeed, one RNA aptamer (Macugen™) directed against vascular endothelial growth factor was approved by the Food and Drug Administration (FDA) in 2005 [8]. An emerging market of aptamers is considered as a rival to antibodies in the biotech industry.

In this article, we selected aptamers ECM-APT-01 and ECM-APT-02 against the epithelial cell adhesion molecule (EpCAM) expressed on primary lung cancer cells isolated from tumors of patients with non-small cell lung cancer (NSCLC) using competitive displacement by monoclonal EpCAM antibody. Moreover, we confirmed aptamers specificity for the EpCAM antibody binding site and its ability to detect EpCAM in clinical samples. EpCAM is a 40 kDa glycoprotein initially identified as a marker for carcinoma, and further studies revealed EpCAM as a prevalent protein also expressed by normal epithelia; however, at a lower level than by carcinoma cells [9]. EpCAM is expressed in various carcinomas such as those in the colon and rectum, prostate, liver, esophagus, lung, head and neck, pancreas, and breast [10]. High-level and mostly homogenous EpCAM expression was found on 85% of adenocarcinomas and on 72% of squamous cell carcinomas [11]. In addition, EpCAM is one of the main biomarkers of circulating tumor cells and microemboli in blood [12].

## 2. Results

### 2.1. Selection of DNA Aptamers to EpCAM Receptor

**Figure 1:**
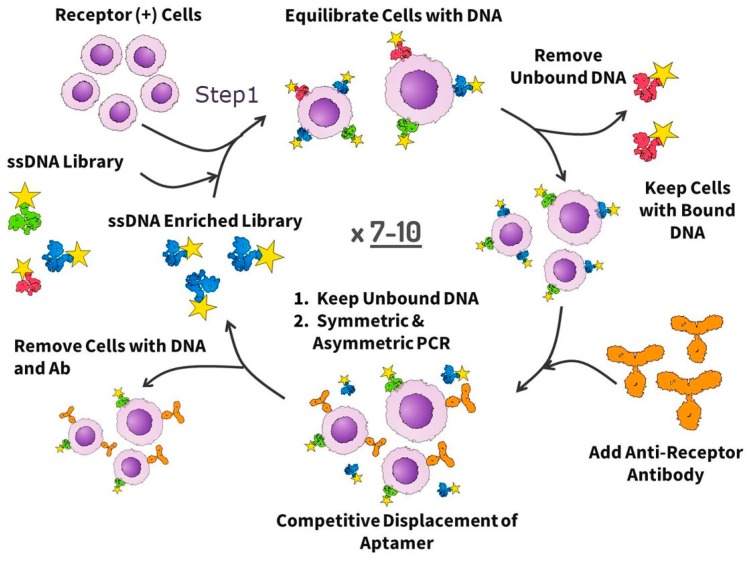
The scheme of DNA aptamer selection using aptamer displacement via antibody. The first several rounds include only positive selection and start with the incubation of the ssDNA library or aptamer pools with receptor positive cells, followed by partitioning unbound DNA, and amplifying bound DNA with symmetric and asymmetric polymerase chain reaction (PCR). In the next rounds, positive rounds alternate with antibody displacement steps and include the incubation of aptamers with the receptor positive cells, washing, the displacement of the bound aptamers by antibodies (Ab), and the following amplification of free aptamers.

To obtain the DNA aptamers against EpCAM, seven rounds of positive selection and two rounds of concurrent replacement by antibodies were performed as shown in Figure 1. In the first selection round, lung cancer (LC) tissues from two patients with different subtypes of squamous LC were used. During the other selection rounds, LC tissue from one patient was used in each round. Different histological types of LC were used in order to increase the probability of selection of aptamers to the EpCAM receptor of different types of LC. Histological types of LC tissues that were used in each selection round are presented in Table 1.

**Figure 2:**
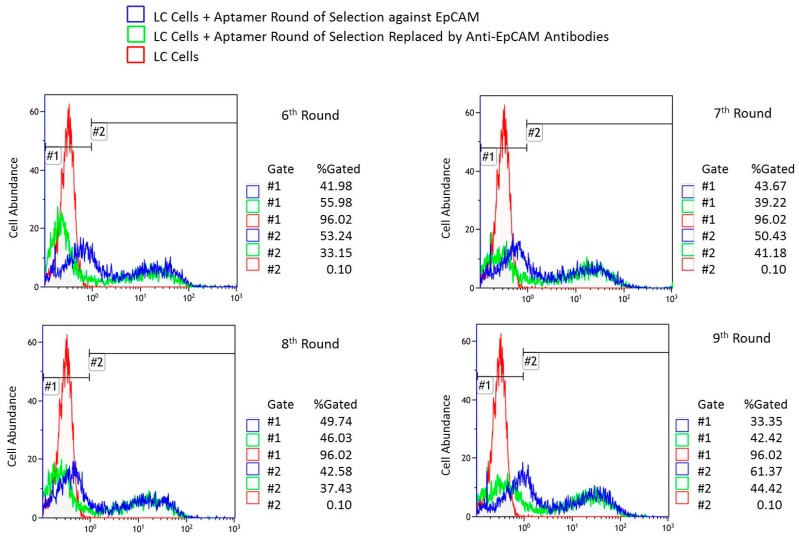
Binding evaluation of aptamer pools. Flow cytometry of lung cancer (LC) cells incubated with pools of 6–9th rounds of aptamer selection against EpCAM at the first step and displaced by EpCAM antibodies at the second step in comparison with LC cells alone.

Overall, nine rounds of aptamer selection against EpCAM^+^ cells were performed. Figure 2 presents flow cytometry results of: LC cells incubated with pools from rounds 6 to 9, the resulting histograms upon addition of antibodies, and the cells alone for comparison. Flow cytometry data showed that round 7 had the best affinity to the LC cells as nearly 19% of LC cells released aptamers into solution after antibody displacement (Table 2). Table 2 summarizes the percentage of LC cells bound with fluorescently labeled aptamers and the percentage of the same cells after its replacement by antibodies as estimated by flow cytometry. For the analyses we used primary cells derived from the whole lung tumor tissues taken after a surgery. The binding as well as the displacement with anti-EpCAM antibodies was not 100%. These tumors are heterogeneous and contain not only cancer cells expressing EpCAM, but also mesenchymal cells, fibroblasts, glandular cells, immune and other blood cells, epithelial cells, necrotic cells, tumor stroma, etc.

Aptamers were sequenced using next-generation sequencing (NGS) technology and their corresponding sequences were analyzed mathematically. During the analysis, unique aptamers forming the greatest families of sequences with good enrichment were chosen according to the algorithm described above. In the end, two leading sequences of the largest families were chemically synthesized (Table 3).

### 2.2. Aptamer Flow Cytometry Analysis

**Figure 3:**
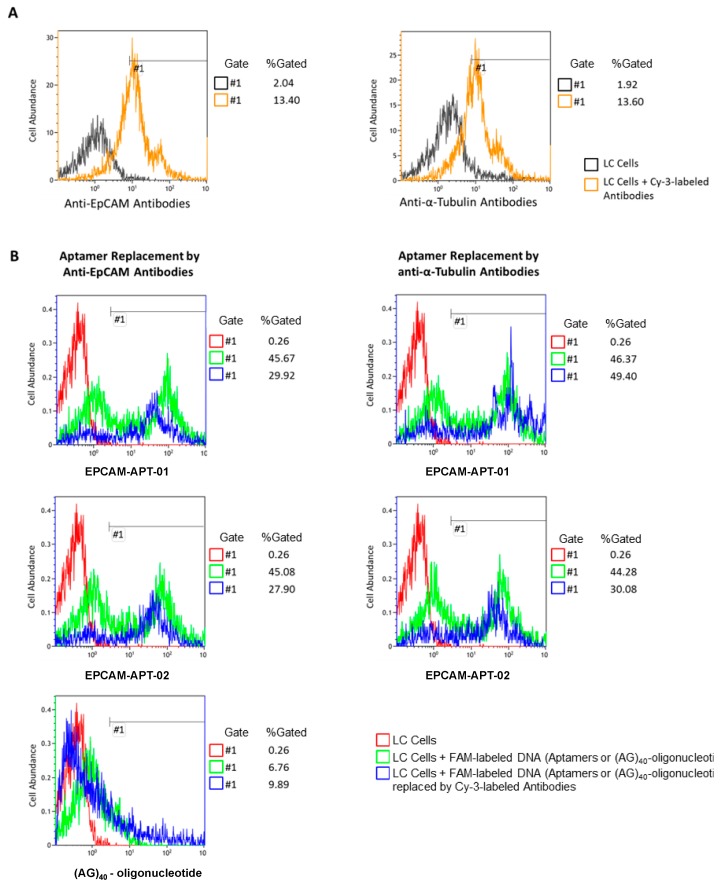
Competitive displacement of aptamers with antibodies. (**A**) Flow cytometry of LC cells and LC cells preincubated with Cy-3 labeled anti-EpCAM or anti-α-Tubulin antibodies. (**B**) Flow cytometry of LC cells (red), LC cells preincubated with 6-carboxyfluorescein (FAM)-labeled EPCAM-APT-01, EPCAM-APT-02 or oligonucleotide (AG)_40_ before (green) and after (blue) replacement by Cy-3 labeled anti-EpCAM or anti-α-Tubulin antibodies.

Aptamers’ affinity to LC cells was estimated using flow cytometry (Figure 3). Replacement of EPCAM-APT-01 by anti-EpCAM antibodies was approximately 15%, while anti-α-Tubulin antibodies did not replace the same aptamer from LC cells (Figure 3B). The partial replacement of aptamer EPCAM-APT-01 may be explained by a lower number of antibody molecules than aptamer molecules bound on the cell surface, hence, the inability of the antibody to replace all the cell surface-bound aptamers. This can be confirmed as only 13% of LC cells were bound with antibodies whereas aptamers stained 45% of cells (Figure 3A,B).

**Figure 4:**
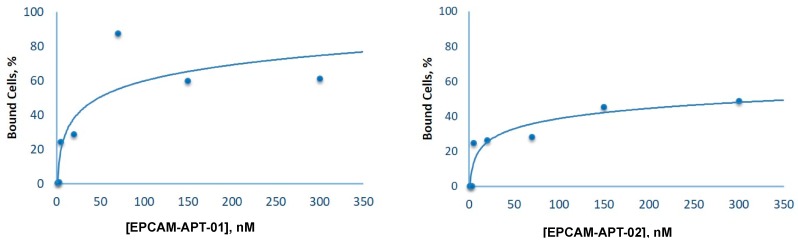
Aptamer affinity curves. The percentage of bound LC cells measured by flow cytometry versus concentrations of EPCAM-APT-01 or EPCAM-APT-02.

Aptamer EPCAM-APT-02 also showed specificity to EpCAM as nearly 18% of LC cells released the aptamer from the surface after replacement by anti-EpCAM antibodies (Figure 3B). Aptamer EPCAM-APT-02 was replaced from cells by anti-α-Tubulin antibodies to a lesser extent than by anti-EpCAM antibodies. This effect may be related to the binding of anti-α-Tubulin antibodies with LC cells leading to a competitive non-specific replacement of aptamer EPCAM-APT-02, that most likely has less affinity to EpCAM than EPCAM-APT-01. Finally, a control sequence, 80 nt oligonucleotide (AG)_40_, was not replaced to any extent by anti-EpCAM antibodies, indicating that it did not bind to EpCAM. The apparent dissociation constants (K_d_) for EPCAM-APT-01 and EPCAM-APT-02 were 25 nM and 15 nM respectively. Figure 4 displays plots of LC cells binding with the corresponding 6-carboxyfluorescein (FAM)-labeled aptamers in concentrations ranging from 1 nM to 300 nM. The relevant flow cytometry data are presented in the Supplementary Figure S1.

### 2.3. Histological Tissue Sections Staining with Aptamers

**Figure 5:**
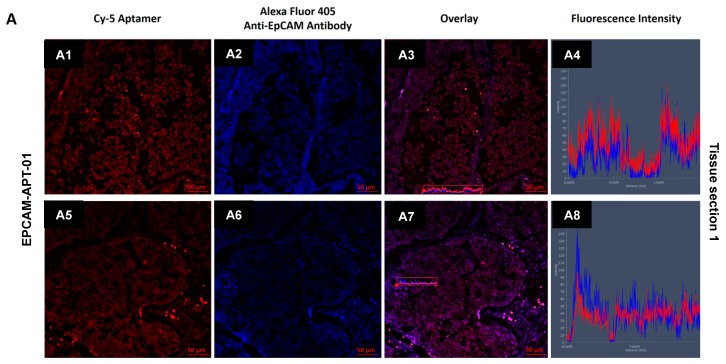

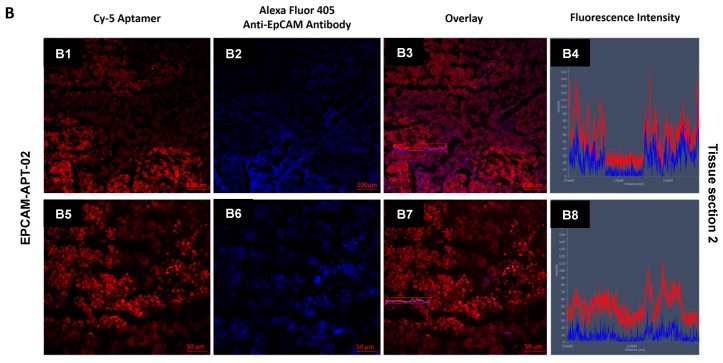
Co-staining aptamers and antibodies. Confocal microscopy of different regions of two squamous LC tissue sections stained with Alexa-Fluor 405-labeled anti-EpCAM antibodies and Cy-5-labeled aptamers EPCAM-APT-01 (**A**) and EPCAM-APT-02 (**B**). (**A1**,**A5**,**B1**,**B5**)—fluorescence of Cy-5-labeled aptamers, (**A2**,**A6**,**B2**,**B6**)—fluorescence of Alexa 405-labeled anti-EpCAM antibodies, (**A3**,**A7**,**B3**,**B7**)—overlays, (**A4**,**A8**,**B4**,**B8**)—overlaid fluorescence intensity spectra from the marked (**A3**,**A7**,**B3**,**B7**)—regions.

Confocal imaging of LC tissue sections simultaneously stained with Cy-5-labeled EPCAM-APT-01 or EPCAM-APT-02 and Alexa Fluor 405-labeled anti-EpCAM antibodies was performed to confirm the binding target of both aptamers (Figure 5). Spectral profile of the tissue sections showed that EPCAM-APT-01 and EPCAM-APT-02 have the same antigen as anti-EpCAM antibodies. Fluorescence intensity analysis of Cy-5-labeled EPCAM-APT-01 and Alexa-Fluor 405-labeled anti-EpCAM antibodies showed co-localization of the two dyes. The fluorescence intensity spectra of both aptamers and anti-EpCAM antibodies match almost completely. EPCAM-APT-02 stained postoperative lung cancer tissues better than EPCAM-APT-01. It can be seen from Figure 5 that the fluorescence intensity spectra of EPCAM-APT-02 higher than fluorescence intensity spectra of anti-EpCAM antibody (Figure 5B8). As for EPCAM-APT-01, its fluorescence intensity spectra is lower than the fluorescence intensity spectra of the anti-EpCAM antibody (Figure 5A8).

### 2.4. Circulating Tumor Cells (CTCs) Isolation Using Aptamers

**Figure 6:**
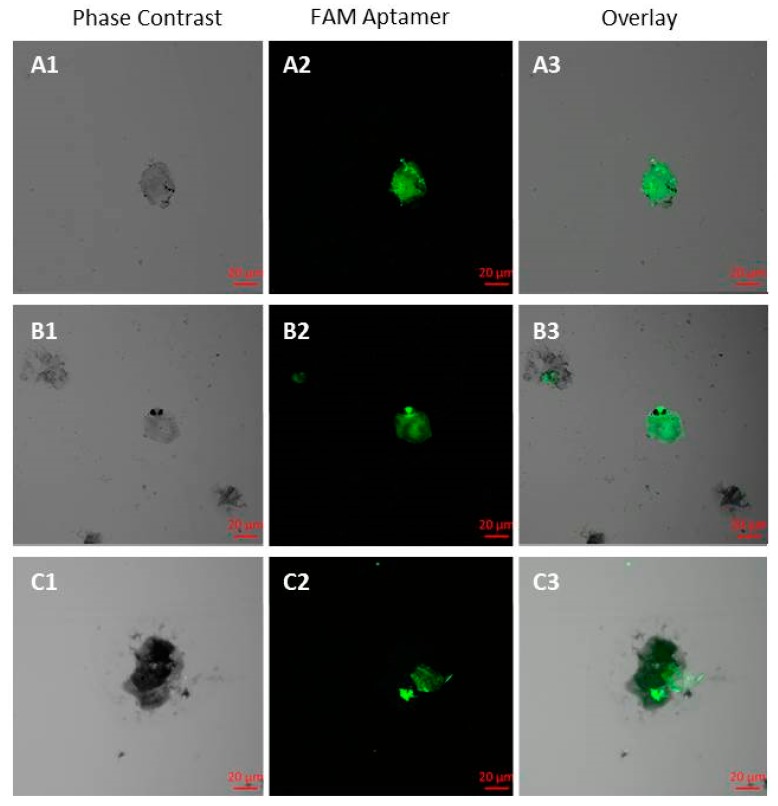
Aptamer-facilitated isolation of circulating tumor cells (CTCs). CTCs were isolated from the blood of two LC patients: ID#101 (**A1**–**A3**) and ID#113 (**B1**–**B3**,**C1**–**C3**), using biotinylated aptamers EPCAM-APT-01 and EPCAM-APT-02 and then stained with the same fluorescent aptamers.

To isolate CTCs from blood, we captured the cells with the biotinylated aptamers EPCAM-APT-01 and EPCAM-APT-02 and streptavidin-coated magnetic beads and then stained with FAM-labeled EPCAM-APT-01 and EPCAM-APT-02 (Figure 6). CTC morphology was confirmed by staining with Romanowsky-Giemsa dye. The number of CTCs isolated by EPCAM-APT-01 and EPCAM-APT-02 varied for each patient from 0 to 6 in 3.5 mL of blood (Table 4). In the blood of NSCLC patients at T1N0M0 (IA) were found 3.0 ± 2.5 CTCs, 2.3 ± 0.5 CTCs at stages T2N2M0, T2N3M0, T3N2M0, T4N0M0 (IIIA), 3.5 ± 0.5 CTCs at stage T4N2M0 (IIIB), and 0.3 ± 0.5 CTCs in blood of patients with non-malignant diseases of the lung. We compared the results obtained by our method with data of other researchers for different patients. EpCAM-based immunomagnetic capture by CellSearch (Veridex) isolated 0.4 ± 0.2 at IIIA stage, 0.4 ± 0.3 at IIIB stage and 6 ± 3.6 IV stage in 7.5 mL of blood of patients with different types of LC [13]. In general, the amounts of CTCs isolated and stained by the aptamers are approximately equivalent to those obtained by the CellSearch system with the help of EpCAM antibodies. For a more accurate comparison a bigger study with more patients involved is required.

## 3. Discussion

Traditionally, aptamers are raised against pure (recombinant) proteins by Systematic Evolution of Ligands by EXponential Enrichment (SELEX) [14,15]. However, it is very likely that at least some of the selected aptamers will not be able to recognize the native proteins in vivo, if (for example) the binding sites are hidden inside a cell, or if the folded structures of recombinant and native proteins differ significantly. The goal of our work was the targeted selection of aptamers to a native cell-surface protein of interest (i.e., a protein that is properly folded, posttranslationally modified, and surrounded by other biomolecules). The selection approach was based on LIgand-Guided Selection (LIGS) of target-specific aptamers [16]. It is a variation of Systematic Evolution of Ligands by Exponential Enrichment (SELEX) and has been introduced by Zumrut et al. in 2016. It does not require any gene transfection and modification of cells. The cells expressing EpCAM were incubated with a DNA library and then washed several times. After removing unbound DNA, the cells were incubated with the excess of a monoclonal antibody against EpCAM. The antibody binds and replaces anti-EpCAM aptamers. Cells are removed and the unbound DNA is amplified by polymerase chain reaction (PCR). All resulted aptamer pools were sequenced, and the aptamer clones from the largest families and the highest enrichment factor were synthesized chemically. Two unique aptamers EPCAM-APT-01 and EPCAM-APT-02 with nanomolar affinity to EpCAM were chosen for CTC detection and isolation.

There is a number of existing DNA aptamers that have been selected to human recombinant EpCAM protein. For example, Wei Duan et al. developed DNA- and RNA-aptamers for EpCAM in 2011 and 2013, respectively [17,18]. As authors mentioned in the second article, RNA is notoriously prone to nuclease degradation, which limits its application in clinical research [18]. DNA-aptamer SYL3C was selected against recombinant EpCAM protein bound with beads. SYL3C specificity and affinity were estimated using flow cytometry and confocal microscopy using different cell cultures.

In some cases, aptamers developed to recombinant proteins do not bind to the same proteins in primary cells and tissues, because these native proteins have posttranslational modifications covering the binding sites of aptamers, and conformations which are not recognizable by the aptamers. In our previous study, DNA aptamers showed good binding ability to postoperative lung adenocarcinoma tissues and had no or low affinity for adenocarcinoma cell cultures [19].

## 4. Materials and Methods

### 4.1. List of Chemicals

Monoclonal antibodies to EpCAM, α-tubulin, a secondary antibody labeled with Alexa Fluor 405 and a secondary antibody labeled with Cy-3 were purchased from Abcam, Cambridge, UK.

### 4.2. Patient-Derived Tumor Samples

This study was approved by the Local Committee on Ethics of the Krasnoyarsk Regional Clinical Cancer Center named after A.I. Kryzhanovsky No. 8/2011 since 16 March 2011 and Krasnoyarsk State Medical University 37/2012 since 31 January 2012, Krasnoyarsk, Russia. Solid tumors were removed aseptically and immediately immersed in ice-cold RPMI-1640 and transported to the laboratory.

### 4.3. Aptamer Selection

The aptamers were selected from an ssDNA library based on the modified cell-SELEX procedure as shown in Figure 1. The selection was started with an ssDNA library containing a 40 nt length variable internal region with three stems and loops and flanked on each side by a 20 nt primer. The final form of the 80 nt library is 5′-CTC CTC TGA CTG TAA CCA CGY ZYZ YZY ZNN NNY ZYZ YZY ZYZ NNN YZY ZYZ YZY ZNN NNY G CAT AGG TAG TCC AGA AGC C-3′ where N is a nucleotide mixture that produces the ratio of A/C/G/T as 1:1:1:1, Y is a mixture that produces the ratio of A/C/G/T as 45:5:45:5, and Z is a mixture that produces the ratio of A/C/G/T as 5:45:5:45. Before each round of selection and binding experiment, the ssDNA library and aptamer pools were denatured by heating for 5 min at 95 °C in Dulbecco’s phosphate buffered saline (DPBS, Sigma-Aldrich, St. Louis, MO, USA) and then renatured on ice for 10 min.

The first five rounds of aptamer development were just positive selections without antibody displacement. The sixth and eighth round included the antibody displacement as follows: (a) incubation of LC tumor tissue with an aptamer pool in DPBS buffer, (b) washing the unbound aptamers with DPBS three times, (c) incubation of the same tumor piece with an anti-EpCAM antibody at the concentration of 2 ng μL^−1^ for 30 min at 22 °C resulting in aptamer replacement by the antibody and release of the bound oligonucleotides into DPBS buffer, (c) removing of the cells with bound antibodies and oligonucleotides by centrifugation and collecting aptamer candidates in DPBS buffer, and (d) amplification of released aptamers by PCR. The seventh and the ninth selection rounds were without an antibody displacement step. In total, nine rounds of selection were performed.

For each round of selection, tumors were separated from necrotic tissues, washed with DPBS and cut into small pieces with a scalpel on a Petri dish with a small amount of DPBS. For the first round of selection, tumor tissue minced into small pieces was incubated with 100 μL of DPBS containing 1 μM ssDNA library for 30 min at 25 °C with gentle shaking. Then, the sample was centrifuged at 4000× *g* for 5 min, supernatant containing unbound aptamers was removed and the pellet was rinsed three times with DPBS. Aptamers bound with tissue were released by denaturation in 10 mM Tris-HCl buffer containing 10 mM ethylenediamine tetraacetic acid (EDTA), pH 7.4 (TE, Sigma-Aldrich, St. Louis, MO, USA) at 95 °C for 10 min followed by centrifugation at 13,400× *g* for 15 min. Next, the supernatant was collected and the aptamers were amplified using symmetric and asymmetric PCR.

For the symmetric PCR, 5 μL of the aptamer pool in 10 mM TE-buffer was mixed with 45 μL of symmetric PCR MasterMix, containing the following: 1× PCR buffer B, 1× Enhancer 1, 1 mM MgCl2, 0.025 U μL^−1^ KAPA2G HotStart Robust polymerase (KAPABiosystems, Wilmington, MA, USA), 220 μM dNTPs, 300 nM forward primer (5′-CTC CTC TGA CTG TAA CCA CG-3′), and 300 nM reverse primer (5′-GGC TTC TGG ACT ACC TAT GC-3′) (Integrated DNA Technologies, Coralville, IA, USA). Amplification was performed using the following PCR program: preheat for 2 min at 95 °C, 15 cycles of 30 s at 95 °C, 15 s at 56.3 °C, and 15 s at 72 °C. Afterward, asymmetric PCR was performed where 5 μL of the symmetric PCR product was mixed with 45 μL of the asymmetric PCR Master Mix containing the following: 1× PCR buffer B, 1× Enhancer 1, 1 mM MgCl_2_, 0.025 U μL^−1^ KAPA2G HotStart Robust polymerase, 220 μM dNTPs, 1 μM FAM-forward primer (5′-FAM-CTC CTC TGA CTG TAA CCA CG-3′), and 50 nM reverse primer (5′-GGC TTC TGG ACT ACC TAT GC-3′). Amplification was performed using the following PCR program: preheat for 2 min at 95 °C, 15 cycles of 30 s at 95 °C, 15 s at 56.3 °C, and 15 s at 72 °C. The PCR product was washed by 30 kDa cutoff filters and the concentration of the ssDNA was measured by NanoDrop (Thermo Scientific, Wilmington, DE, USA). Fluorescence of ssDNA labeled with FAM was analyzed in the gel-documenting system GBOX/EF2-E (Syngene, Frederick, MD, USA). Evolved aptamer pools were stored at −20 °C.

The sixth and the eighth rounds of aptamer selection were done as described below. The asymmetric PCR product obtained from the previous round was incubated with lung cancer tissue for 30 min with shaking at 25 °C. Thereafter, the sample was incubated with monoclonal antibodies to EpCAM (2 ng μL^−1^) for 30 min with shaking at 25 °C. As a result of this process, aptamers bound to the cell membrane receptors were replaced by antibodies and released into DPBS. The sample was centrifuged at 4000× *g* for 5 min and the supernatant was collected. The aptamers were then amplified using symmetric and asymmetric PCR and washed by cutoff filters. The seventh and the ninth rounds were positive selections and similar to the first five rounds.

### 4.4. Flow Cytometric Binding Analysis of Aptamers

The affinity and specificity of the evolved aptamers was defined by flow cytometry using FC-500 Flow Cytometer (Beckman Coulter Inc., Porterville, CA, USA). Lung cancer material was washed with Dulbecco’s Phosphate Buffered Saline (DPBS) and minced into small pieces and then pipetted gently with DPBS to remove cell clusters and obtain a homogeneous solution. Cell suspension was filtered through 70 μm filters; obtained cells were centrifuged at 3000× g for 5 min and washed three times with DPBS. Next, cells were pre-incubated with masking DNA (1 ng μL^−1^ of salmon sperm DNA) for 30 min and then with 50 nM of FAM-labeled aptamers from each pool of a selection round or synthetic aptamer sequences for 30 min at 25 °C with shaking. Each sample contained 3 × 105 cells. LC cells pre-incubated with 1 ng μL^−1^ masking DNA and 50 nM FAM-labeled (AG)40-oligonucleotide were used as a control. The measurements were carried out using flow cytometry. The samples were then incubated with 2 ng μL^−1^ of Cy-3 labeled anti-EpCAM monoclonal antibody for 30 min with shaking at 25 °C followed by flow cytometry.

To evaluate the dissociation constants of EPCAM-APT-01 and EPCAM-APT-02, LC cells were incubated with 1 nМ, 3 nМ, 5 nМ, 20 nМ, 70 nМ, 150 nМ, and 300 nМ of FAM-labeled aptamers. The measurements were carried out using flow cytometry. The data were analyzed with Kaluza^®^ 1.2 Software (Beckman Coulter Inc., Indianapolis, IN, USA). The dissociation constants were determined from the plots as half of the concentrations observed at maximum binding.

### 4.5. NGS Sample Preparation Procedure

To barcode the pools for NGS, the pools were first prepared by adding an 8 nt barcode to the 5′ end of the aptamers using symmetric PCR. Approximately 10 ng in 5 µL volumes of each pool was used for a PCR reaction in order to amplify and barcode single-stranded (ss) DNA molecules using a forward primer (5′-BBB BBB BBC TCC TCT GAC TGT AAC CACG-3′) containing an 8-base barcode (B) at the 5′ end and a reverse primer (5’-GGC TTC TGG ACT ACC TAT GC-3′). Symmetric amplification was performed by preparing five 50 µL PCR reactions using the following: 7% DMSO, 1.5 mM MgCl_2_, 0.2 mM dNTP (Promega Corporation, Madison, WI, USA), 0.4 µM barcoded forward primer, 0.4 µM reverse primer, and 0.02 U µL^−1^ of Phire II polymerase. PCR was performed in a Mastercycler pro S thermal cycler (Eppendorf, Hamburg, Germany). The settings for the thermal cycler were as follows: melting at 94 °C for 30 s, annealing at 58 °C for 15 s and extending at 72 °C for 15 s. After PCR amplification, the five 50 µL aliquots for each pool were combined and then separated and visualized by gel electrophoresis (2% agarose, 1× Tris-Acetate EDTA buffer). The band corresponding to 88 bp DNA was cut-out, then sliced into small pieces, weighed and placed in a new 50 mL Eppendorf tube with 1 mL of water per 1 mg of gel and left to elute for 24 h. The buffer was then collected and the DNA was concentrated using 3 kDa centrifugal filters (Pall Corporation, Show Low, AZ, USA). All the pools were combined and sequenced at Eurofins Genomics (Louisville, KY, USA).

### 4.6. Mathematical Analysis of Aptamer DNA Sequences

The central 40-nucleotides random region of the 80 nt aptamer sequences was analyzed. The 20-nucleotides forward and reverse primers were dropped in the analysis to simplify the procedure. The main challenge of the mathematical analysis was to choose unique aptamers forming the best families of sequences with good enrichment. To solve this problem, the following algorithm was used.

For each aptamer a from the best round of aptamer selection against EpCAM the total amount of copies of the similar sequences $f_{40}^{\gamma }\left(a\right)$ where γ=0, 1,…, 7 and $f_{20}^{\gamma }\left(a\right)$ where γ=0,1,2,3 was calculated.

Aptamers with the greatest values of $f_{40}^{\gamma }$ and $f_{20}^{\gamma }$ with various values of γ were chosen (i.e., aptamers forming the largest families of sequences).

From the aptamers that were chosen in paragraph 2, sequences with the best enrichment were chosen (r>1 in most rounds of selection).

The detailed description of the mathematical analyses is presented in Appendix A.

### 4.7. Histological Tissue Staining

Tissue pieces were frozen in liquid nitrogen and sliced into 5 µm sections by Microm HM525 Cryostat and placed on poly-lysine coated glass slides. First, nonspecific binding of the antibodies was blocked by incubation of the sections with 10% of Bovine Serum Albumin (Sigma-Aldrich) for 30 min, followed by incubation with primary anti-EpCAM antibody (2 ng μL^−1^) in a humidified atmosphere for 1 h and then with a secondary antibody labeled with Alexa Fluor 405 (2 ng μL^−1^) in a humidified atmosphere for 1 h and then washed three times with DPBS. Nonspecific binding of the aptamers was blocked with yeast RNA (1 ng µL^−1^) (Sigma-Aldrich) and then incubated with 50 nM of Cy-5-labeled aptamers for 1 h in a humidified atmosphere and washed with DPBS. Bio Mount mounting medium (Bio-Optica, Milano, Italy) was used to fix the sections.

### 4.8. Isolation of CTCs from Human Blood

Isolation of CTCs was performed from 3.5 mL of patients’ blood 1–1.5 h after collecting into Becton, Dickinson Vacutainer Heparin Tubes (Becton, Dickinson and Company, Franklin Lakes, NJ, USA). The blood was centrifuged (1500× *g* for 10 min) to remove the plasma. The cell pellet was transferred into a 15 mL centrifuge tube using a bovine serum albumin (BSA) treated tip. Red blood cells (RBCs) were lysed with hypotonic NH_4_Cl solution in a Vacutainer tube. Cells were rinsed with 2 mL of 0.42% NH_4_Cl with heparin, pipetted up and down 5 times and poured into the 15 mL tube with 8 mL of 0.42% NH_4_Cl with heparin, incubated for 10 min on a shaker and centrifuged at 3500× *g* for 5 min. The remaining cell pellet was resuspended in 100 μL DPBS and incubated with masking yeast RNA (0.1 mg mL^−1^) for 30 min at room temperature to reduce nonspecific binding. The sample was centrifuged at 3500× *g* for 5 min, the supernatant was removed and incubated for 30 min with 100 µg of streptavidin-coated paramagnetic beads (Promega Corporation) functionalized with 100 nM biotinylated aptamers against tumor cells. The cells bound with the magnetic particles via the aptamers were separated on a magnetic stand, and resuspended in 100 μL of calcium and magnesium free DPBS buffer and concentrated by a magnet. The pellet containing mostly CTCs was stained with FAM-labeled aptamers at a final concentration of 50 nM for 30 min. Cell smears were prepared for quantification of CTCs in blood of patients. After staining, to prepare the smears, the cell pellet was spread evenly on a glass slide and then fixed in methanol for 5 min, followed by staining with Romanowsky-Giemsa dye. The counting of circulating tumor cells was done on a fluorescent microscope Axiostar plus (Carl Zeiss Group, Oberkochen, Germany).

## 5. Conclusions

We presented a targeted selection of DNA aptamers to a native EpCAM based on LIGS and obtained the aptamers concurrently replaced by anti-EpCAM antibody. The resulting aptamers were successfully applied for histological tissue staining of NSCLC and isolation and detection of circulating tumor cells in clinical samples of the peripheral blood of patients with different histological types of lung cancer and benign tumors of lung, bronchus and fibrosarcoma of the pleural cavity. The number of aptamer-detected CTCs correlates closely to the number of CTCs in the blood of LC patients isolated with the EpCAM-based immunomagnetic CellSearch system.

The developed aptamer clones, EPCAM-APT-01 and EPCAM-APT-02, may be further used for EpCAM detection in real clinical samples in most human adenocarcinomas and it is a marker for cancer stem cells in several solid cancers. However, before using EpCAM aptamers for clinical diagnostics, it is necessary to study epitope structures of EpCAM-aptamer complexes by affinity mass spectrometry, protein X-ray crystallography, and small-angle X-ray scattering [20,21].

## Figures and Tables

**Table 1:** Histological types of lung cancer tissues used in the selection of aptamers against epithelial cell adhesion molecule (EpCAM).

Selection Round	Lung Cancer Type	Lung Cancer Stage	Specification of Selection Round
1	Squamous keratinizing LC Squamous LC	T4N3M0 T3N1M0	Positive
2	Moderately differentiated squamous LC	T1N0M0	Positive
3	Moderately differentiated squamous LC	T2N0M0	Positive
4	Adenocarcinoma	T3N2M0	Positive
5	Adenocarcinoma	T3N2M0	Positive
6	Squamous LC	T3N2M0	Ab displacement
7	Squamous LC	T3N1M0	Positive
8	Squamous LC	T3N0M0	Ab displacement
9	Squamous LC and adenocarcinoma (mixed type)	T3N2M0	Positive

**Table 2:** A comparison of the percentage of LC cells bound with fluorescently labeled aptamers against the percentage of the same cells after aptamer replacement by antibodies as estimated by flow cytometry.

Aptamer Target	Pool Number	LC Cells Bound with Aptamers, %	LC Cells Released Aptamers after the Concurrent Replacement, %
EpCAM	6	53.24	18.09
	7	50.43	19.25
	8	42.58	5.15
	9	61.37	16.95

**Table 3:** DNA sequences of anti-EpCAM aptamers.

Aptamer Name	Aptamer Sequence (Primer Regions Shown in *Italic* and Unique Region Shown Bold)	Enrichment (*r*)					Number of Copies in Round 7	Total Number of Sequences in Round 7
		2->3	3->4	4->5	5->6	6->7		
EPCAM-APT-01	5′*CTCCTCTGACTGTAACCACG***ACACGCACAAATGTCAGTGTACCGCACTCGCACATTCTTA***GCATAGGTAGTCCAGAAGCC*3′	2.4	1.2	1.2	1.4	1.5	44	160,415
EPCAM-APT-02	5′*CTCCTCTGACTGTAACCACG***GTGCGCGTACCACCATGTGTACACACTGCATGTTTGGTTA***GCATAGGTAGTCCAGAAGCC*3′	3.1	1.3	1.1	1.5	1.4	42	160,415

**Table 4:** Amounts of CTCs isolated from the blood (3.5 mL) of LC patients and patients with non-malignant diseases of the lung using aptamers EPCAM-APT-01 and EPCAM-APT-02.

Number	Patient ID	Diagnosis	Histological Type	Lung Cancer Stage	# of CTC
1	97	LC	Low-differentiated squamous carcinoma	T1N0M0	1
2	101	LC	Adenocarcinoma	T1N0M0	6
3	120	LC	Adenosquamous carcinoma	T1N0M0	2
4	113	LC	Adenosquamous carcinoma	T2N2M0	3
5	92	LC	Low-differentiated squamous carcinoma	T2N3M0	2
6	91	LC	Low-differentiated squamous carcinoma	T3N2M0	2
7	124	LC	Squamous carcinoma	T4N0M0	2
8	104	LC	Squamouscarcinoma	T4N2M0	4
9	123	LC	Squamous carcinoma	T4N2M0	3
10	93	Benign tumor of the mediastinum	N/A	N/A	0
11	96	Fibrosarcoma of the pleural cavity	N/A	N/A	1
12	122	Benign tumor of bronchus and lung	N/A	N/A	0

## Supplementary Materials

The following are available online at https://www.mdpi.com/2072-6694/11/3/351/s1, Figure S1: Binding of individual aptamers to cells.

## Appendix A

### Mathematical Analysis of Aptamer DNA Sequences after NGS

We present a pool of aptamers P as a multiplicity of words in the alphabet {A, T, G, C}. Each word ω∈P is characterized by the length |ω| and by the number of copies in the pool f(ω). We indicate the fact that a word ϑ is a subword ω as ϑ⊂ω.

To estimate degree of proximity of two words $\omega_{1}$ and $\omega_{2}$ with the same length we use Hamming distance $d\left(\omega_{1},\omega_{2}\right)$ i.e., the number of positions where the relevant symbols are different. To estimate degree of proximity of two words ω_1_ and ω_2_ we use the following function:

$g\left(x,\omega_{1},\omega_{2}\right)=min_{\vartheta_{1}\subset \omega_{1},\vartheta_{2}\subset \omega_{2}}\left\{d\left(\vartheta_{1},\vartheta_{2}\right):\left|\vartheta_{1}\right|=\left|\vartheta_{2}\right|=x\right\},$ where $x\in \{1,\;2,\;\ldots ,\min\left(\left|\omega_{1}\right|,\left|\omega_{2}\right|\right)\}$.

Notice, that $g\left(x,\;\omega_{1},\;\omega_{2}\right)=0$ when there are the same subwords with the length x in the words $\omega_{1}$ and $\omega_{2}$.

Let us take a as an aptamer from the pool P. For $x\in \left\{1,\;2,\;\ldots ,\;min_{\omega \epsilon P\;}\left\{\left|\omega \right|\right\}\right\}$ and γ≥0:

$$U_{x}^{\gamma }\left(a\right)=\left\{\omega b\in P:g\left(x,a,b\right)\leq \gamma \;\gamma \right\}.$$

A multiplicity of aptamers $U_{x}^{\gamma }\left(a\right)$ contain aptamers from pool P which have subwords with the length x close to subwords a when the values of γ are small. It is natural to consider aptamers from a $U_{x}^{\gamma }\left(a\right)$ as a family of aptamer a in pool P. The value $f_{x}^{\gamma }\left(a\right)=\underset{\omega \epsilon U_{x}^{\gamma }\left(a\right)}{\sum }f\left(\omega \right)$ characterizes the size of a family.

We denote the part of aptamer a in pool P as s(a,P): $s\left(a,P\right)=\frac{f\left(a\right)}{\sum_{\omega \epsilon P}f\left(\omega \right)}$. If there are two pools $P_{1}$ and $P_{2}$ and aptamer a contained in both, we call a number $r=\;\frac{s\;\left(a,\;P_{2}\right)}{s\;\left(a,\;P_{1}\right)}$ as enrichment of a during the transition from $P_{1}$ to $P_{2}$. It is noticed [21] that the significant value of r for the pair of consistent selection rounds demonstrates in most cases the great potential of an aptamer to bind with a target.
