# Supplementary material for: Development of DNA Aptamers to Native EpCAM for Isolation of Lung Circulating Tumor Cells from Human Blood
Source: Cancers (Basel). 2019 Mar 12;11(3):351. doi: 10.3390/cancers11030351 (PMC6468627; doi:10.3390/cancers11030351)
Supplement: Supplementary file 1 [file cancers-11-00351-s001.pdf]

Supplementary Materials: Development of DNA Aptamers to Native EpCAM for Isolation of Lung Circulating Tumor Cells from Human Blood

Galina S. Zamay, Olga S. Kolovskaya, Tatiana I. Ivanchenko, Tatiana N. Zamay, Dmitry V. Veprintsev, Valentina L. Grigorieva, Irina I. Garanzha, Alexey V. Krat, Yury E. Glazyrin, Ana Gargaun, Ivan N. Lapin, Valery A. Svetlichnyi, Maxim V. Berezovski and Anna S. Kichkailo

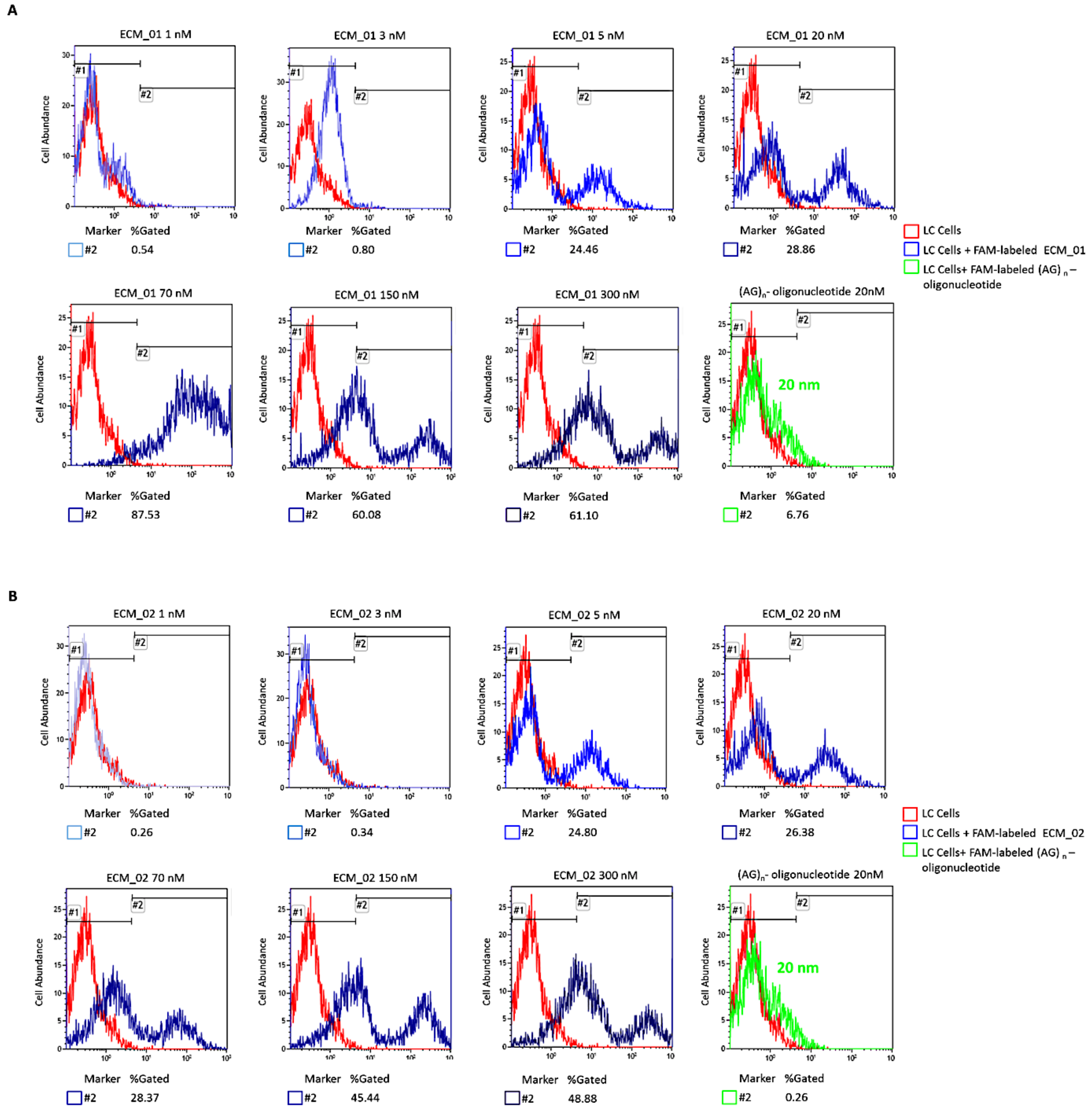

Figure 1. Binding of individual aptamers to cells. (**A**) Flow cytometry of LC cells and LC cells preincubated with FAM-labeled ECM_01 in concentrations 1 nM, 3 nM, 5nM, 20 nM, 70nM, 150 nM and 300 nM or FAM-labeled $(AG)_{40}$-oligonucleotide in concentration 20nM. (**B**) Flow cytometry of LC cells and LC cells preincubated with FAM-labeled ECM_02 in concentrations 1 nM, 3 nM, 5nM, 20 nM, 70nM, 150 nM and 300 nM or FAM-labeled $(AG)_{40}$-oligonucleotide in concentration 20nM.

© 2019 by the authors. Licensee MDPI, Basel, Switzerland. This article is an open access article distributed under the terms and conditions of the Creative Commons Attribution (CC BY) license (http://creativecommons.org/licenses/by/4.0/).
